# Supplementary material for: Are carbon emissions trading and green financial instruments synergistic? -Comprehensive quantitative research based on content analysis
Source: PLoS One. 2024 Mar 7;19(3):e0298601. doi: 10.1371/journal.pone.0298601 (PMC10919601; doi:10.1371/journal.pone.0298601)
Supplement: S1 Data — (ZIP) [file pone.0298601.s001.zip › Data/地级市Z_F工作报告.zip!/╣π╢1⁄2/╒┐╜¡╩╨/└┤╫╘╣1⁄2╓┌║┼í╛┬φ┐╦╩2╛▌═°í┐_2012.docx]

各位代表：

　　我代表市人民政府向大会作政府工作报告，请予审议，并请市政协委员和其他列席的同志提出意见。

　　2011年及本届政府任期工作回顾

　　2011年是实施“十二五”发展规划的第一年。一年来，在市委的正确领导下，在市人大及其常委会和市政协的监督支持下，市政府坚持以科学发展观为统领，紧紧围绕做大做优经济“蛋糕”和争当粤西地区振兴发展龙头的目标，深入实施“工业立市、港口兴市、生态建市”发展战略，加快转变发展方式，努力建设幸福湛江，全面完成了市十二届人大第七次会议确定的各项任务，实现了“十二五”良好开局。

　　一、综合经济实力稳步提升

　　预计全市完成生产总值1650亿元，增长13%，增速高于全省3个百分点；其中海洋产业增加值增长18.9%,占GDP比重31%。人均生产总值23000元，增长11.5%。产业结构继续优化，三次产业比重由21︰41︰38调整为19︰44︰37。预计地方财政一般预算收入80.03亿元，增长26.44%；来源于湛江的财政总收入450亿元，增长32%；完成税收363亿元，增长40.3%。

　　投资拉动有力。预计完成固定资产投资700亿元，增长32.9%；其中重点项目完成投资226.5亿元。寄托湛江五年崛起希望的特大型钢铁、石化项目取得突破性进展，宝钢湛江钢铁有限公司正式成立，配套重件码头、砂石料码头交工验收，原料码头、自备电厂和厂东区开工建设；中科炼化项目获得国家核准并正式动工。晨鸣林浆纸一体化、奥里油电厂油改煤、粤电生物质发电、湛江港至廉江输油管道、南三大桥等9个项目竣工；第十四届省运会主场馆、湛江港宝满集装箱码头一期、霞山港区散货码头、茂湛铁路、鉴江供水枢纽和雷州青年运河节水改造工程等20个项目加快建设；粤西再生能源基地等8个项目开工；湛江机场迁建、湛江港40万吨级航道、大唐雷州电厂、京信东海岛电厂、30万吨燃料乙醇等项目前期工作顺利推进。

　　工业后劲增强。预计规模以上工业总产值1660亿元,增长17%；增加值570亿元,增长14%。工业对经济增长的贡献率超过40%。中海油南海西部公司油气产量超过1000万方，东兴炼油厂产量突破500万吨大关，分别成为我市年产值超300亿元企业；龙腾球团项目完成产量156万吨、产值18.3亿元，均创投产以来新高；晨鸣林浆纸一体化项目完成产量17.9万吨、产值9.42亿元。编制实施《湛江市产业与产业园区布局规划》；湛江石化产业园和冠豪高新东海岛特种纸产业基地开工建设；广州（湛江）产业转移工业园被认定为全省循环经济工业园和产业转移重点园区，并获全省综合评分第一名。民营经济预计完成增加值800亿元，增长15%，占GDP比重达到48.5%。

　　农业平稳发展。预计农业总产值558亿元，增长6.9%。糖蔗总产量1065.3万吨、北运菜总产量226万吨、水产品总产量107万吨，均居全省首位。正大（湛江）现代农业综合开发项目、南国花卉科技园顺利推进，国联水产境外投资项目获国家发改委批准。开发补充耕地9.12万亩，耕地保有量增加到730.6万亩，连续三年获得省政府奖励。增加农村土地流转50万亩。新增4个省级、1个国家级农业标准化示范区，新增3家省级、5家市级农业龙头企业和375家农民专业合作社。湛江农垦现代农业龙头示范作用进一步增强。建设海洋与渔业自然保护区18个，建成硇州国家级中心渔港和省级特呈岛深水网箱产业园。基本完成集体林权制度改革，森林覆盖率28.5%。

　　服务业较快增长。预计服务业增加值610亿元，增长14%。港口吞吐量1.55亿吨，增长14%。社会消费品零售总额800亿元，增长18%；君豪酒店、民大广百湛江店建成营业；家电下乡累计零售额66.7亿元，增长21.7%。物业服务企业发展到120多家，营业收入近2亿元。金融机构本外币存款余额1657.25亿元，比年初增长5.9%；贷款余额826.34亿元，比年初增长14.57%；存贷比51.3%。徐闻被列为省旅游综合改革示范县；廉江成功创建省级旅游强县；特呈岛被命名为全国特色景观旅游名村示范单位；预计全年接待游客1812.3万人次，增长28.1%；旅游业总收入90.2亿元，增长38.2%。

　　外经贸快速回升。预计外贸进出口总额42亿美元，增长24%，其中出口20.15亿美元，增长19.4%，增速均列全省第一。水海产品加工与小家电制造成功晋升首批国家和省外贸转型升级示范基地。合同外资3.13亿美元，增长3.3倍；实际利用外资4700万美元，增长55.1%。

　　二、城市建设扎实推进

　　完成“三环四通”交通规划编制；湛江大道、官南公路和调顺岛跨海大桥“两路一桥”前期工作顺利推进；东海岛西南跨海通道项目列入省“十二五”交通规划。基本建成东海岛疏港公路、奋勇大道、乐金路、滨湖路二期工程，完成汉口路、东堤路、东海大道改造和海湾大桥西连接线工程股份重组。建成霞山污水处理厂二期、吴川污水处理厂二期工程和麻章污水处理厂，动工建设东海岛污水处理厂，市区生活污水处理率88.36%。完成海田路景观整治和三岭山森林公园整治一期工程，基本完成滨湖公园一期工程，增加公园用地1.1万亩，建成绿道40公里，新增绿地面积超100万平方米，市区环境空气质量优良率100%，继续名列全国环保重点城市前列，入选当年中国十佳低碳生态城市。

　　三、人民生活持续改善

　　全年预计用于民生的财政投入125.62亿元，增长17.14%，占地方财政一般预算支出的70.49%。城镇居民人均可支配收入17371元，增长13.5%；农民人均纯收入8150元，增长18%，增速创13年来新高。加强物价调控，新增平价商店100家，预计全年CPI涨幅5%左右。城镇新增就业6.9万人，农村劳动力新增转移就业15万人。新型农村养老保险参保105万人，超额完成省下达任务；职工基本医疗保险和城乡居民基本医疗保险参保率分别达98%和98.4%。建设廉租房518套2.87万平方米、公租房2384套14.3万平方米。改造农村贫困群众危房1.68万户。扶贫开发“双到”工作累计投入帮扶资金15亿元，建成项目1810个，85%贫困村贫困人口和95%贫困村稳定脱贫。

　　四、社会事业全面进步

　　编制完成湛江市《中长期教育改革发展规划纲要》和《学前教育三年行动计划》；中小学校舍安全工程超额完成省下达任务；学前三年入园率80.85%；高中阶段毛入学率88.93%；高考上线人数居全省前三位；职业教育学生毕业4.4万人，就业率98.4%；民办学校发展到514所，在校生17.57万人；市四中、市八小、开发区一中新校区动工。新增9家国家级高新技术企业，认定3家省级、16家市级自主创新企业；实施高新技术项目105项，其中获得科研成果奖51项；专利申请量和授权量分别达到1000件和826件，增长22%和8%。成功承办首届全省农民文艺大汇演和举办雷州历史文化展；建成农家书屋607家；新增省级非物质文化遗产项目10个；市档案馆新馆开工奠基，市档案局被评为全国先进集体。完善运用现代商业保险资源参与城乡居民医保一体化管理服务的“湛江模式”，受到温家宝总理等中央领导批示肯定。启动9项基本和6项重大公共卫生服务项目，城市社区卫生服务覆盖率98%；创建省卫生村31条；湛江中心人民医院迁建认捐2.3亿元并开工奠基；获评全国无偿献血先进城市。农村信息化试点通过省验收。人口计生工作跨入全省先进行列。第四次被评为“全国双拥模范城”。参加第26届世界大学生运动会、第七届全国城市运动会取得优异成绩，成功举办第五届海上国际龙舟邀请赛和市第十二届体育节。

　　五、民主法制建设成效显著

　　完成“五五”普法规划，积极实施《法治湛江建设五年规划》。全年办理省市人大代表议案、建议和政协委员提案276件，办复率100%。规范性文件报备率、行政复议和行政执法投诉结案率达到100%。强化审计职能，为国家增收节支3419万元，避免经济损失1.43亿元。市人民来访接待厅和网上信访厅建成启用，社会矛盾纠纷调处成功率92.9%。加强应急预案演练，妥善处置各类突发事件和重大涉稳案件。强化安全生产专项整治，有效防范和遏制重特大事故。严厉打击走私活动，连续9年没有出现暴力抗拒缉私和群体性走私案件。深入开展群防群治和严打整治行动，连续8年被省评为综治工作优秀市。

　　各位代表！过去一年各项工作任务的全面完成，标志着本届政府确立的五年施政目标顺利实现。五年来，全市人民团结一心，众志成城，艰苦拼搏，完成了市十二届人大各次会议确定的任务，实现了生产总值、规模以上工业增加值、服务业增加值、海洋产业增加值、民营经济增加值、固定资产投资、社会消费品零售总额、旅游业总收入、税收收入和地方财政一般预算收入等“十个翻番”，经济实力显著提升，发展后劲显著增强，城市面貌显著改观，人民生活显著提高。这是应对挑战、攻坚克难的五年，是着眼长远、夯实基础的五年，是推进科学发展、建设幸福湛江成果丰硕的五年。五年历程不同寻常，五年成绩来之不易，这是中共湛江市委正确领导的结果，这是全市人民团结奋斗的结果，这是社会各界鼎力支持的结果。在此，我代表市人民政府，向各级人大代表和政协委员，向全市广大工人、农民、知识分子、干部职工，向驻湛人民解放军、武警官兵、公安干警和各民主党派、人民团体、社会各界人士，向长期关心支持我市发展改革的港澳同胞、台湾同胞、海外侨胞、国际友人，表示崇高的敬意和衷心的感谢！

　　在充分肯定成绩、增强发展信心的同时，我们也清醒认识到我市经济社会发展存在的主要问题和困难：一是投资总量不足，工业发展速度不快，利用外资水平不高；二是城市发展滞后，建设资金筹措难度加大，城市管理亟待加强；三是民生事业发展和社会管理水平有待提高；四是投资环境有待进一步改善；五是政府机关作风和干部干事创业精神与新时期、新形势、新任务的要求还有差距，突出表现在敢于担当意识不强、开拓创新锐气不足、破解难题办法不多、工作落实力度不够，等等。我们对此必须高度重视，采取更加有力措施加以解决，努力把各项工作做得更好。

　　新一届政府任期奋斗目标和2012年工作安排

　　新一届政府任期的五年，是湛江蓄势突破、后发崛起、进位赶超的关键时期。新起点谋划新伟业，新湛江踏上新征程，新一届市政府肩负承前启后、继往开来的历史使命，承载全市人民的信任、重托和期待，必须带领全市人民同心同德，奋力拼搏，开拓进取，朝着全力推动五年崛起、加快建设幸福湛江的新目标奋勇前进。

　　新一届政府任期的奋斗目标：实现经济崛起。到2016年，全市生产总值达到4000亿元，年均增长15%；地方财政一般预算收入220亿元，年均增长22.5%；全港吞吐量达到3亿吨；工业总产值达到5500亿元，年均增长16%，基本建成以钢铁、石化和造纸为龙头的现代产业体系。实现城市崛起。按照省委省政府提出的“五个城市”和我市“五城同创”要求，2013年建成全国卫生城市，2014年建成国家环保模范城市，2015年建成国家生态园林城市，2016年创建国家低碳发展示范城市和全国文明城市取得重大进展，初步建成具有集聚力、辐射力和引领力的区域性国际城市。实现生态崛起。全市森林覆盖率达到30%以上，城市建成区绿化覆盖率达到46%；万元GDP能耗比2011年下降17%；城市生活污水处理率达到90%以上，市区环境空气质量继续位居全国环保重点城市前列。实现文化崛起。建成全省及环北部湾地区的文化强市、科教强市、人才强市，文化产业增加值占生产总值的比重达到5%。实现民生崛起。全面建设小康社会取得突破进展，城乡居民收入大幅度增长，生活质量显著提高。

　　2012年，是新一届市政府工作的起始之年，也是我市重大项目加速推进、基础设施加速建设、发展潜能加速释放之年。做好今年的经济社会发展工作，意义重大。

　　2012年我市经济社会发展的总体要求是：以邓小平理论和“三个代表”重要思想为指导，以科学发展观为统领，以做大做优经济“蛋糕”为头等大事，全面贯彻落实市第十次党代会精神，加快实施“工业立市、港口兴市、生态建市”发展战略，坚持“四个第一”，改善“四大环境”，坚定不移促发展，全力以赴抓招商，凝心聚力上项目，实实在在惠民生，为实现五年崛起奠定坚实基础。

　　2012年我市经济社会发展的预期目标是：生产总值增长13%，人均生产总值增长11%；固定资产投资增长20%以上；港口吞吐量增长10%；社会消费品零售总额增长18%；消费价格涨幅控制在4%左右；外贸进出口总额增长7%，其中出口增长7%；实际利用外资增长15%；地方财政一般预算收入增长15%，人均财政一般预算收入增长13.5%；城镇居民人均可支配收入增长11%，农村居民人均纯收入增长12%；城镇登记失业率控制在3.5%以内；人口自然增长率控制在7.36‰以内；市区生活污水处理率90%，城镇生活垃圾无害化处理率45%；万元生产总值能耗下降3.66%；完成省下达的节能减排等约束性指标。

　　2012年必须着力抓好的重点工作：

　　一、以扩大投资为主导，进一步提升发展动力

　　全力实施以钢铁石化为龙头的重点项目。2012年安排重点项目64项，总投资2280.55亿元；其中年内建成项目7个、在建项目21个、开工项目19个、开展前期工作项目17个，计划完成投资294.5亿元。举全市之力抓好钢铁、石化、林浆纸三大工程，动工建设湛江钢铁项目，加快建设中科炼化项目，分别完成投资50亿元；推进晨鸣林浆纸一体化项目二期工程，力争年内动工。建成100万吨渤海菜籽油综合加工、广东半岛科技集团麻章生产基地等项目；开工建设京信120万千瓦东海岛电厂、中能酒精30万吨燃料乙醇、湛江国家石油储备库和东海岛港区杂货码头等项目；力争大唐雷州电厂动工；加快湛江机场迁建、湛江港40万吨级航道等项目前期工作。

　　全力实施“三环四通”交通工程。全面启动市区成环、海湾成环、半岛成环，市外通高铁、县县通高速、镇镇通快速、村村通公路的“三环四通”大交通工程，推动交通骨架成形、公路成网、港口成群，形成大港口阵容、大交通格局、大城市框架、大开放气势。坚持统一规划、分步实施、分级负责、各级联动，迅速掀起交通建设大会战高潮。年内完成交通项目投资25亿元，建成东海岛疏港公路，加快茂湛铁路和海湾大桥西连接线公路建设，动工建设东海岛铁路、东海岛跨海大桥复线、环雷州半岛一级公路徐闻海滨段和雷州环城大道东段，加快合浦至湛江铁路和汕头至湛江、东海岛至雷州、吴川至廉江高速公路前期工作。

　　全力实施“十百千”投资计划。在全市“五大五新五特”产业、交通市政基础设施、房地产等十几个领域和行业，引导数百名民营企业家投资超千亿元。进一步密切企业家的联系，尊重企业家的地位，支持企业家的事业，关怀企业家的亲人，维护企业家的尊严，激发企业家感恩家乡、回报桑梓的投资愿望和行动。进一步放宽准入条件、放开投资领域，鼓励本地民营企业扩大投资，大张旗鼓、满腔热情欢迎外地湛江籍民营企业家带着资金、带着项目、带着感情回乡投资创业，力争年内民营固定资产投资超过200亿元。

　　全力推进央企省企对接合作。充分发挥我市得天独厚的深水大港、区位交通、土地资源和生态环境优势，主动对接央企省企，促进湛江五年崛起。建立与央企省企更加紧密的战略合作伙伴关系与互动协调机制，引导更多央企省企参与发展先进制造、港口物流、金融服务、旅游休闲等现代产业，参与港口、交通、城市建设，参与企业改制、资产重组，加快产业转型升级。积极发挥市国资公司的国有资产进退平台作用，加强与央企省企战略合作。大力支持宝钢集团、中石化、中海油、中石油、中航油、中外运、招商局、中铁建、中远集团、中海集团、中粮集团、中交集团、中纺集团、华润集团、大唐集团和粤海集团等大型央企省企在湛江拓展投资领域，加大投资强度，扩大投资规模。举办央企投资合作洽谈会。

　　全力推进招商融资。实施积极的财政政策，强化银政、银企合作，推动融资模式和产品创新，加快筹集发展资金。充分发挥市基投集团、交投集团、水务集团等融资功能，注重有效利用土地、滩涂、港口、岸线等资源，鼓励采用BT、BO、BOT、独资、合作等形式，促进资源变成资本。完善财税体制，落实“谁招商、谁受益”，充分调动县（市、区）招商积极性。每月轮流举行县（市、区）招商融资签约活动，确保月月有签约、县县有项目、年年有投产。以欧美为方向、港澳为重点，精心筹办系列经贸招商活动，引进更多资本实力雄厚的国外战略投资者；积极支持在湛外商投资企业增资扩产。年内全市引进内资500亿元，力争实际利用外资6000万美元以上。

　　全力推动全民创业。制定促进全民创业政策，健全服务平台，设立扶持奖励基金，为全民创业提供贷款担保、贴息和补贴，引导广大群众关注实业、投资实业、发展实业，实现实业致富。树立一批成功创业典型，倡导“劳动光荣、致富光彩、创业英雄”的社会风尚，鼓励大中专毕业生自立创业、有业人员再创新业、科技人员带头创业、待业群体自主创业、打工者转化创业和外地客商投资创业，形成百姓创家业、能人创企业、干部创事业的生动局面，以全民创业推动全市投资大突破、经济大发展。

　　各位代表！五年崛起看湛江，扩大投资是关键。大投资大发展，快投资快发展。投资看招商，招商看项目。我们一定要坚持发展第一、工业第一、招商第一、项目第一，以投资扩总量、优结构、创优势、增后劲，让大投资提振发展信心，让大投资助推湛江腾飞，让大投资给力五年崛起！

　　二、以构建现代产业体系为支撑，进一步提升发展竞争力

　　推进建设海洋经济发展示范区。坚持海洋先行，落实《广东省海洋经济综合试验区发展规划》，大力推进临港工业、港口物流、海洋能源、滨海旅游、海洋运输、海洋油气、海洋渔业、海产品加工、海洋制药、海洋工程等产业全面协调发展。坚持陆海统筹，建立一批海洋经济重点园区，培育一批海洋经济产业基地，壮大一批海洋经济骨干企业，支持开发区申报省级海洋综合开发试验区，构建“蓝色产业”集聚带。坚持政府主导，按照“五个用海”要求，编制全市海洋经济综合发展规划，启动东海岛南部100平方公里填海造地工程；鼓励多元投资主体兴建公用码头；完善乌石等国家级中心渔港建设；探索海洋旅游资源开发模式，重点在南三岛、特呈岛等地发展休闲旅游、度假酒店、会议中心及文化创意等产业。坚持校地合作，依托广东海洋大学等高校，加强海洋人才培育，加快海洋科技与产业创新。

　　推进区域经济错位发展。认真落实《湛江市产业与产业园区布局规划》，深入开展招商引资“三讲三评”活动，加强产业协调和政策引导，发挥优势，错位发展，壮大县域经济。开发区要紧抓钢铁、炼化等重大项目建设契机，进一步提升劲头、当好龙头，成为湛江大工业发展的主战场和经济发展的排头兵。赤坎区要重点打造风情老街区、开拓建设“北出口”、规划控制调顺岛、美化净化两江河，努力建成“首善之区”。霞山区要大力发展商业、商务、商港“三商”产业，成为粤西消费中心，打造魅力之区。麻章区要发挥“黄金通道、交通枢纽、生态走廊、教育基地”优势，打造亮丽“西城新区”。坡头区要主打东岸板块，建设海东新区，打造湛江海湾城市新名片。雷州市要按照生态发展要求，建设工业重镇、农业强市和文化名城。廉江市要紧抓珠三角产业转移、湛江重大项目带动、北部湾经济区崛起三大机遇，率先建成北部湾新兴工业城市。遂溪县要重点发挥毗邻中心城市和交通枢纽优势，建成湛江市后花园。吴川市要重点发挥民营资本优势，建设富裕幸福的江海城市。徐闻县要重点发挥三面环海优势，加快推动城市走向海岸、产业对接海南，建设滨海度假旅游海峡城市。奋勇经济区要重点发挥区位、土地和华侨优势，创新体制机制，培育发展“五新”产业集聚区，建成继东海岛之后我市又一经济增长极。

　　推进建设现代农业强市。落实粮食工作政府负责制，抓好现代标准农田建设和政策性水稻保险，稳定粮食种植面积。坚持依靠农垦、农业龙头企业、农民专业合作社和科技带动，广泛运用良种良法，提升农业产业化水平。规划建设农垦湖光农场万亩连片农业产业化示范中心区，推进正大（湛江）现代农业综合开发项目和吴川黄坡万亩基本农田保护示范区、遂溪杨柑万亩火龙果产业园、雷州万亩大棚西瓜基地建设，打造现代农业示范样板。做大做强水产饲料业，创建深水网箱养殖示范区。发展畜牧业标准化养殖。建设300万亩桉树林基地，完成造林20万亩。大力发展水利事业，年内鉴江供水枢纽工程具备供水能力，加快雷州青年运河节水改造工程；完成37宗病险水库除险加固和2宗海堤加固达标工程；启动南渡河治理等水利工程前期工作。

　　推进发展现代服务业。实施现代服务业提升计划，培育壮大服务型经济。积极发展滨海旅游业。主动策应和对接海南国际旅游岛，规划建设以南三岛、特呈岛开发为重点的“五岛一湾”滨海旅游产业园，重点实施霞山渔人码头等16个投资超亿元旅游项目，完成投资45亿元；加快建设特呈岛度假会议中心、南三岛和南屏岛旅游区及吴川吉兆湾、琼州海峡北岸等滨海旅游集聚区；推进湖光岩风景区创建国家5A景区。积极发展特色餐饮业。开展海鲜美食十大名菜、十大名店、十大名厨评选活动，规划建设滨湖海鲜美食风情街，擦亮“中国海鲜美食之都”品牌。积极发展商贸物流业。建成中央直属食糖储备库等3个总投资14亿元的商贸项目，动工建设湛江商贸物流城等8个总投资48亿元的商贸项目；培育霞山水产品批发市场、南方国际水产交易中心等4个大型专业市场；做大做强港口物流产业。积极发展地方金融业。推动统筹城乡发展金融改革创新综合试验区建设，健全城乡金融服务体系；启动建设中央商务区，力争年内2家企业上市融资，引进中外银行业和保险机构4家以上、小额贷款机构2家以上；组建2家村镇银行。积极发展房地产业。扩大普通商品住房有效供给，扶持大型城市综合体建设，推动房地产市场健康发展。积极发展对外贸易。设立外贸转型升级专项基金，重点发展壮大国家级水海产品和省级小家电转型升级示范基地，规划建设东海岛海关特殊监管区，扩大外贸进出口。

　　推进发展战略性新兴产业。以省级湛江高新技术产业开发区为重点，积极培育发展“五新产业”，创建科技创新创业孵化基地，努力培植一批高端产业集群，带动工业由大变强。规划发展智能家电、生物制药等产业基地，推进冠豪高新东海岛特种纸产业基地、双林医药生产基地建设。加快雷州、徐闻等一批风电场建设，打造广东最大风电基地。组织实施新兴产业科技专项，培育文化创意、数字出版、移动多媒体、动漫游戏等新兴产业，启动建设国家光盘生产示范基地和文化创意产业园区。推动湛江省级高新区升级国家高新区、奋勇经济区申报湛江高新区延伸区。

　　各位代表！五年崛起看湛江，产业发展是重点。产业成就大业，产业决定事业。我们一定要坚持规划引领，强化产业导向，统筹产业协调，壮大产业集群，让现代产业之力夯实工业强市之基，让现代产业之花喜报五年崛起之春，让现代产业之光照亮幸福湛江之路！

　　三、以建设区域性中心城市为抓手，进一步提升辐射带动力

　　强化龙头引领提升城市规划水平。加快编制湛江市《城乡发展战略规划》和《城市总体规划》，实现与土地利用规划、主体功能区规划、海洋经济综合发展规划、经济社会发展规划“五规合一”。推进分区规划编制。加快规划信息化建设，提高城市规划执行效能。大力培养和引进一批具有国际视野的城市规划师、城市建筑师和城市设计师，注重研究城市历史，借鉴成功经验，塑造城市形象，铸就城市文化，形成具有热带风貌、滨海特色、中西合璧的城市建筑风格，提升海湾城市品位。

　　强化功能配套提升城市建设水平。建成疏港大道二期和开发区龙平北路、永平路等13条道路；推进新湖大道、海东快速干线、明政路等6条道路建设和海滨大道、洪屋路改造；开工建设“两路一桥”及社坛路、南方路和东海岛岛东大道、岛南大道、通港大道等19条道路；打通体育北路；完成振兴路、源珠东路前期工作。启动建设市新行政中心。筹划市区交通枢纽中心。在市区人流和交通量大的地段规划建设人行天桥或地下通道。从今年开始，三年完成城区骨干道路沥青化改造，以崭新城市面貌迎接第十四届省运会召开；年内实施海北路、椹川大道、绿塘路、康顺路、康宁路、跃进路和中山一路等路面改造。加快建设三岭山森林公园综合整治及生态恢复二期、滨湖公园二期工程和银帆公园；动工建设园林垃圾生态处理场；新建一批小游园、小绿地；扎实抓好东菊公园、麻章公园、赤坎水库公园、坡头南调公园等项目前期工作。开展城市雕塑设计建造。规划建设一批停车场。完成城市公共自行车系统一期工程。推进城市景观亮化工程。建设东海岛、坡头、麻章水厂和金沙湾观海长廊一期排水工程，改造城区地下排水系统，解决雨天“水浸街”问题。积极推动城中村、城郊村、旧城区和旧厂房等重点地块集中连片改造，打造新的城市功能区。

　　强化责任落实提升城市管理水平。推进和实现城管执法重心下移，形成以区为主体、职能部门为保障、街道（社区）为基础、市民人人参与的城市管理体系。强化属地管理，落实城管区长责任制。集中整合社会资源，探索实施城市卫生管理市场化运作。加快城市管理数字化建设，完善城市综合管理考评制度，实现管理规范化、科学化。修订出台《查处违法建设工作过错责任追究办法》，坚决遏制违法建设。以“创卫”、“创模”为动力深入开展城市环境综合治理，重点整治城市“六乱”；突出解决非法营运和交通拥堵问题，创建良好的交通环境。

　　强化生态优先提升城市宜居水平。既要经济崛起，又要蓝天碧水。全面构建沿海、江河两岸和铁路、高速公路、国道省道两侧生态景观林带和防护林带，力争年内成功创建“广东省林业生态市”。推进新湖大道、疏港大道二期等道路配套绿化及市区绿道二期工程，大力建设城市绿道网。加大饮用水源和跨行政区流域水质综合监测与保护力度，加强鹤地水库等水资源管理，严厉打击河道、库床非法采砂、取水等行为；开工建设合流水库扩容和东海岛红星水库扩容工程，加快赤坎水库功能调整。深化海洋生态保护，坚持公共资源公共化，顺应城市发展和人民群众的强烈要求，上半年完成湛江港湾非法渔业设施及碍航物清理整治，还海于景、还海于城、还海于民，让湛江港湾重归自然、再现美丽。加快寸金渠和绿村河治理。基本完成生活垃圾处理场三期工程，动工建设市区垃圾焚烧发电厂，改造提升一批公厕，建设一批建筑垃圾处置场和垃圾中转站。建成坡头、东海岛污水处理厂，完成坡头、麻章污水处理厂截污管网工程，动工建设赤坎污水处理厂三期工程和开发区平乐污水处理厂。深入实施资源、环保价格和生产要素改革，从严控制主要污染物排放总量，完成省下达的减排任务。推进名镇名村示范村和新民居建设，打造生态宜居新农村，新增市级以上卫生村100条。扎实抓好“五城”同创，年内创建国家卫生城市达到申报条件，创建国家环保模范城市取得重大进展。

　　各位代表！五年崛起看湛江，城市建设是名片。我们一定要坚持城市有特色、景观有个性、建筑有文化、管理有章法，加快建设“六大中心”，致力把城市实力做强、功能做优、形象做美、品位做高，打造宜居宜业宜游的区域性国际城市，让湛江成为人人向往的繁荣之地、创业之都、幸福之家！

　　四、以发展社会事业为载体，进一步提升民生保障力

　　加快建设文化湛江。推进社会主义核心价值体系建设，弘扬“胸怀理想、艰苦奋斗、开放兼容、勇立潮头”的湛江人精神，大力开展文明创建和评比活动。把握文化发展新机遇，着力建设一批文化设施，培育一批文化人才，打造一批精品力作。完成市、县（市、区）两级经营性文化事业单位和国有文艺团体转企改制。加快建设市档案馆新馆，动工建设湛江新闻大厦、市文化艺术中心，规划建设湛江歌剧院。加强文化遗产保护，举办市第八届文化艺术节和粤西（湛江）文化节，办好首届湛江海洋图书文化节，打响“雷州文化”品牌，努力提升文化软实力，要让文化繁荣，不能让文化贫穷！

　　加快发展社会事业。推动义务教育均衡发展和普通高中教育优质化、多样化、特色化建设，不断满足群众教育需求；创建2个全省教育强区、18个教育强镇；建设市区幼儿园11所和村级幼儿园178所；建成市四中新校区，完成职教基地整体规划，动工建设湛江机电学校、市第二技工学校和湛江幼儿师范高等专科学校新校区；积极推进教育信息化工程；支持驻湛高校扩大办学规模。继续扶持建立省、市级工程技术研究开发机构；建设国家海珠及其制品质检中心、省家电产品和镇级农产品检测站。出台引进培养高层次人才政策，采取多种形式引入一批急需和紧缺人才。有序推进第14届省运会各项筹备工作，年内完成省运会主场馆建设工程量50%；完善并开放金沙湾海滨浴场；力争“国家跳水训练基地”落户，筹建跳水运动学校；抓好湛江籍运动员参加2012年伦敦奥运会有关工作；办好第六届海上国际龙舟邀请赛和市第十三届体育节。深入开展计划生育优质服务县（市、区）创建活动，统筹人口发展。加强国防后备力量建设。切实抓好编制、统计、外事、侨务、民族、宗教、对台、地震、人防、老区、粮食、供销社、气象、社会科学和妇女儿童等工作。

　　加快保障和改善民生。完善各级人力资源公共服务平台，加强就业培训、援助和服务。关心农民工就业与生活，建设“农民工工资无拖欠城市”。推进社会保险扩面征缴，实现城乡居民社会养老保险全覆盖。深化医疗价格改革，巩固广覆盖多层次的医疗保障体系；支持发展民营医疗机构；加快建设湛江中心人民医院新院，动工建设广东医学院附属医院新院和市120医疗急救指挥中心，筹建市第一中医院新院和市妇幼保健院新院。充分发挥住房公积金保障作用，有效解决干部职工住房难问题；确保教师保障房用地，扩大教师公租房建设；建成钢铁项目搬迁村民安置小区。逐步提高全市公务员津贴标准和教师工资福利待遇，落实公务员有计划带薪休假制度。建立物价上涨与低收入群众临时价格补贴联动机制。深入推进扶贫开发“双到”工作，完成扶贫年度任务。

　　加快创新社会管理。设立社会管理工作机构，抓好社会组织培育发展，形成“党委领导、政府主导、社会协同、公众参与”的社会管理格局。推进县、镇、村三级综治信访维稳中心(站)建设，确保群众利益诉求表达畅通，强化行政复议解决行政纠纷主渠道作用，健全社会稳定和重大事项风险评估机制，做到情况掌握在基层、问题解决在基层、矛盾化解在基层。完善应急指挥平台网络和应急疏散避护场所，提高突发事件预防、预警和应急处置能力。设立知识产权举报投诉服务中心。探索流动人口和特殊人群网格化、信息化管理服务新模式。落实安全生产“一岗双责”，深化安全隐患排查治理。严格依法依规加强食品药品生产、流通、消费各个环节监管，让人民群众吃得安全、吃得放心。加快社会治安视频监控系统、各级监控中心平台、交警智能指挥中心和省（粤西）反走私与海防电子监控中心建设，严厉打击涉黑、涉枪、涉毒、涉赌和“两抢一盗”等各种违法犯罪活动，增强缉私能力，维护国家安全和社会稳定，保障广大群众安居乐业。

　　办好18件民生实事。完成城区38个农贸市场升级改造；完成北桥河和赤坎江整治工程，动工南柳河整治工程；城镇新增就业6.5万人、失业人员实现再就业4万人，组织旅游企业提供就业岗位3000个；完成农村劳动力免费技能培训4万人，新增转移就业13万人；建设乡镇（街道）公共就业保障服务平台119个；加快210所中小学规范化建设，动工建设中等职业学校项目16个、30.7万平方米；建成乡镇（街道）文化站21个、农村文化室370个；投入1000万元建设10间乡镇卫生院；市区新增公交车50辆、出租车300辆；完成农村公路硬底化改造850公里；建设城镇保障性安居工程3631套，着力解决低收入家庭住房困难；改造农村低收入困难群众住房1.68万户；完成33条贫困村1056户“整村搬迁”；新建水库移民新村85条，解决4000户、1.8万人住房困难；新建和改扩建乡镇敬老院（中心）20所；完成21宗农村饮水安全工程，解决28万人饮水安全问题；创建10个综合减灾示范社区；扶持建设农副产品生产基地10个、储备基地4个，新增平价商店100家。

　　各位代表！五年崛起看湛江，改善民生是考验。我们一定要把保障与改善民生作为政府工作的出发点和落脚点，做到民有所呼，必有所应；民有所难，必有所助；民有所忧，必有所虑；民有所求，必有所为，努力为民建基业、为民谋福祉，让全市人民共享发展成果、共度美好生活，让全市人民更加幸福、更感自豪、更有尊严！

　　五、以加强政府自身建设为根本，进一步提升执行力

　　着力提高依法行政水平。推进实施“六五”普法规划和《法治湛江建设五年规划》。深入推进政务公开，完善政府新闻发布、政府信息公开和公共企事业单位办事公开制度。健全科学民主决策机制，完善专家咨询论证、社会公示听证和网络问证等制度。全面推行重大行政处罚备案制和行政执法责任制。自觉接受人大及其常委会的依法监督，认真执行各项决议，定期报告工作，规范性文件审查率、备案率达到100%。积极支持人民政协履行职能。做好人大代表建议和政协提案办理工作。鼓励新闻监督，接受新闻监督，宽容新闻监督。

　　着力转变作风抓落实。湛江五年崛起，时不我待，必须只争朝夕、真抓快干。千难万难，干就不难，千招万招，干是实招。开展“机关作风整治年”活动，大力倡导“五荣五耻”创业观，坚决问责办事拖拉、执行落实不力行为。构建层级之间、岗位之间的责任链条，做到事有专管之人、人有明确之责、责有限定之期，确保重要工作、重点项目、重大事项定一件、干一件、成一件。充分发挥督查督办职能，让督办有地位、有威信、有效果，促进各项工作落到实处、抓出实效、干出实绩。

　　着力改善四大投资环境。围绕全力以赴上项目、全民动员抓招商、全心全意帮企业的要求，着力改善政务、诚信、治安和交通四大投资环境，让投资者放胆投资、放手创业、放心发展。改善政务环境，关键是心，心到手到！人人都是政务环境，人人都是投资环境，要全心全意为人民服务，诚心实意为投资者服务。建成新政务服务中心并加挂投资服务中心牌子，开展争创群众满意窗口、优质服务品牌、优秀服务标兵活动；进一步精简各类审批事项，市审批权限范围内重点项目审批提速率达到80%以上；全面清理各种行政事业性收费，努力为投资者提供“零障碍、低成本、高效率”服务。改善诚信环境，关键是德，德有信有！政府答应的条件、承诺的优惠不兑现，是对投资者、对老百姓最大的伤害，必须强化诚信政府建设，做到言而有信、诺而则行、行而必果，严格落实《湛江市鼓励招商引资若干优惠政策》；大力营造全民讲诚信的浓厚氛围，每一个干部、每一个公民、每一个企业都要以诚为本、严守信用。改善治安环境，关键是公，公在安在！坚持重典治乱，秉公执法、公正司法，平等保护各类市场主体合法权益，维护社会公平正义；注重解决影响群众安全和投资发展的突出治安问题，特别对各种黑恶势力操纵控制市场的欺行霸市、非法垄断等犯罪行为要重拳出击、露头就打，同时坚决依法查处其在政府部门和公职人员中的“保护伞”。改善交通环境，关键是路，路通财通！加快完善雷州半岛交通体系，全面实现高速公路、国道出入口与县（市）公路和港口、工业园区、旅游区、重点市场无缝连接，形成半岛对内交通一体化、对外交通快捷化。各级政府各部门务必高度自觉、主动作为，全力提升软环境，全面改善硬环境，让湛江成为众望所归的发展热土、百凤还巢的投资宝地、千鸟归林的创业乐园。

　　着力推进体制改革创新。改革出动力，创新出活力。创新政府管理服务模式，进一步理顺政府与市场、企业、社会的关系，推进政企、政资、政事、政府与市场中介组织分开，凡是能由市场机制调节的事，坚决放给市场；凡是可由社会组织解决的事，舍得向社会组织“放权”。深化市场经济体制改革，促使政府和各级干部更加注重运用市场经济手段调控经济、管理社会、解决问题、推动发展。进一步明晰区级管理职能，逐步下移管理重心，下放管理权限，下倾财税利益，形成权责一致、分工合理、运转协调的市与区行政管理体制。

　　着力加强廉政建设。完善惩治和预防腐败体系，认真落实党风廉政建设责任制，大力运用科技手段，提升廉政风险防控效能，强化对关键人员、关键岗位、关键环节的监督和制约，进一步拓展从源头上防治腐败工作领域。深化领导干部任期经济责任和政府投资项目审计，公开审计结果。加强政府工作人员廉政教育，提升境界，弘扬正气，坚守底线，自觉拒腐防变。加大案件查处力度，严厉惩处腐败行为，以反腐倡廉的实际成效取信于民。

　　各位代表！五年崛起看湛江，干部队伍是中坚。豪情满怀，理想是第一源泉；干事创业，干部是第一要素。我们一定要坚持“为民、务实、清廉”，努力建设对人民负责、为人民尽职、让人民满意的百姓政府，让政府成为人民雨中的大伞、路上的驿站、心里的靠山！

　　各位代表！湛江五年崛起的进程既宏伟壮丽，又充满艰辛。但是，任何困难都不能改变湛江人民甩掉欠发达地区帽子、创造幸福生活的强烈愿望，任何挑战都不能动摇湛江人民发愤图强、进位赶超的坚定信心，任何障碍都不能阻挡湛江人民后发崛起、争当龙头的豪迈步伐。让我们在中共湛江市委带领下，励奋进之志，聚和谐之力，鼓实干之劲，在推动湛江五年崛起的新征程中，采撷新的希望，书写新的篇章，创造新的辉煌！
